# A Metabolomic Approach to the Study of Wine Micro-Oxygenation

**DOI:** 10.1371/journal.pone.0037783

**Published:** 2012-05-25

**Authors:** Panagiotis Arapitsas, Matthias Scholz, Urska Vrhovsek, Stefano Di Blasi, Alessandra Biondi Bartolini, Domenico Masuero, Daniele Perenzoni, Adelio Rigo, Fulvio Mattivi

**Affiliations:** 1 Department of Food Quality and Nutrition, Research and Innovation Centre, Fondazione Edmund Mach, San Michele all'Adige, Italy; 2 Department of Computational Biology, Research and Innovation Centre, Fondazione Edmund Mach, San Michele all'Adige, Italy; 3 Consorzio Tuscania, Firenze, Italy; 4 Department of Biological Chemistry, University of Padova, Padova, Italy; University of California Davis, United States of America

## Abstract

Wine micro-oxygenation is a globally used treatment and its effects were studied here by analysing by untargeted LC-MS the wine metabolomic fingerprint. Eight different procedural variations, marked by the addition of oxygen (four levels) and iron (two levels) were applied to Sangiovese wine, before and after malolactic fermentation.

Data analysis using supervised and unsupervised multivariate methods highlighted some known candidate biomarkers, together with a number of metabolites which had never previously been considered as possible biomarkers for wine micro-oxygenation. Various pigments and tannins were identified among the known candidate biomarkers. Additional new information was obtained suggesting a correlation between oxygen doses and metal contents and changes in the concentration of primary metabolites such as arginine, proline, tryptophan and raffinose, and secondary metabolites such as succinic acid and xanthine. Based on these findings, new hypotheses regarding the formation and reactivity of wine pigment during micro-oxygenation have been proposed. This experiment highlights the feasibility of using unbiased, untargeted metabolomic fingerprinting to improve our understanding of wine chemistry.

## Introduction

Exposure of wines to excessive amounts of oxygen causes irreparable damage, leading to the production of off-flavors associated with aldehydes and bacterial spoilage. For centuries wine quality has been associated with freshly fermented grape juices, since man lacked the technology to preserve wine under anaerobic conditions. In ancient times the Egyptians already produced terracotta jars which could be hermetically sealed, probably representing the first true containers for fermented juices [1]. Pasteur was the first to point out the influence of the amount of oxygen on the organoleptic development of red wines during maturation and the importance of fine tuning [2]. The effect of oxygen on the sensorial quality of red wines has been recognised to be typically hormetic, being clearly positive at low levels and negative above a certain level. In recent times research has led to the development of modern devices suitable for finely tuning the amount of oxygen reaching the wine, now widely used and of considerable economic interest, particularly for the production of red wines. However, we now realise that there is a significant need to make such technology more efficient. One answer is to improve our understanding of chemistry in order to develop biomarker sets that can be measured and reported at appropriate levels, in order to gain control over this process.

Micro-oxygenation is a common winemaking practice for red wine, consisting of the continuous addition of small amounts of oxygen to wine in order to improve its colour, aroma, texture and conservation. In terms of sensorial qualities, the micro-oxygenation process increases fruity and spicy flavours, enhances the stability of red tones and decreases herbaceous aromas and astringency [3]–[6]. Chemically, this technique increases both volatile compounds such as acetaldehyde, vanillin and syringaldehyde, and the non-volatile products of reaction between anthocyanins and flavanols, pyruvic acid, acetaldehyde and vinylphenols. Moreover, it has been proved that micro-oxygenation influences the concentration of different secondary metabolites in wine [3], [5]–[11]. Metals, such as iron or copper, play an important catalytic role in these reactions, since they can act as electron donors when there is a small amount of oxygen present and with typical wine pH. Studies have concluded that different flavonoids, such as anthocyanins and catechins, but also acetaldehyde, are sensitive to the addition of iron to wine [12], [13]. In recent years there has been increased interest in the literature as regards target analysis directed at the study of known reaction mechanisms occurring during micro-oxygenation, before and after malolactic fermentation, or at finding new mechanisms [3], [5], [6], [8]–[16].

This is certainly a difficult and highly complex task, since the molecules present in wines include a large number of primary metabolites (sugars, amino acids, organic acids, lipids, etc.) and secondary metabolites (phenolics, alkaloids, sterols, lignans, terpenes, fatty acids, etc.); which can derive from grapes, forming during alcoholic and malolactic fermentations or during wine ageing [17].

Past approaches to metabolite analysis include metabolite profiling, metabolite fingerprinting, and target analysis [18]. By their nature, these approaches provide a restricted and less than comprehensive view of the metabolome and as a result, only a very small fraction of the metabolome is analysed. The idea of metabolomics (an untargeted approach) is to analyse as many metabolites and different compound classes as possible in a single chromatographic run.

Lately, a few studies using untargeted approaches to evaluate the influence of oak barrels, the geographical origin of grapes, the year of harvest, the grape variety and the yeasts and bacteria responsible for alcoholic and malolactic fermentation in wine have been published [19]–[29] Liger-Belair et al. studied the untargeted chemical fingerprint of champagne aerosols [29]. The instrumentation used for most of these studies consisted of GC/MS and/or NMR instruments or direct injection mass spectrometry (DIMS) and only Cuadros-Inostroza et al. and Vaclavik et al. used LC/MS. Nevertheless, both studies focused on discrimination of red wines based on raw material differentiation [19], [25]. All these studies have demonstrated that restricted target analysis of specific metabolites misses a large part of the molecular information regarding the metabolome of the wine and that untargeted metabolomics can be a powerful tool for the molecular fingerprinting of a complex beverage such as wine. At all events, the use of targeted data analysis of experiments carried out using untargeted techniques which have also provided interesting results in the field of wine should not be underestimated [30]–[32].

The scope of this work was to use UPLC-QTOF MS to produce a detailed, untargeted picture of the changes caused by the addition of oxygen and metal to the metabolic fingerprint of a Sangiovese wine following micro-oxygenation. The doses of oxygen added with micro-oxygenation were those typically used in wineries and the levels of metals were within the limits usually present in wines, without any extreme values, in order to obtain the most realistic possible results, which would be most directly relevant in commercial and technological terms. This was a constraint due to the scale of the experiment, in order to prevent the depreciation of wine, and was also our preferred choice since the use of extreme values is considered inappropriate for the study of biphasic effects [33].

To our knowledge, this is the first paper applying metabolomic analysis to wine micro-oxygenation.

## Results and Discussion

Before data processing, analytical system stability was examined. The chromatograms of a standard mix (STDMIX) were used for visual inspection and fast scrutiny of the data. We first evaluated extracted ion chromatograms for each analyte and these gave identical, non-distinguishable peaks in the different chromatograms in terms of size, shape and retention time. Mass spectra were also identical thus fulfilling the criteria. Average RSD was c. 0.1% for the retention times and c. 5% for the peak areas.

### Number of features

**Figure 1:**
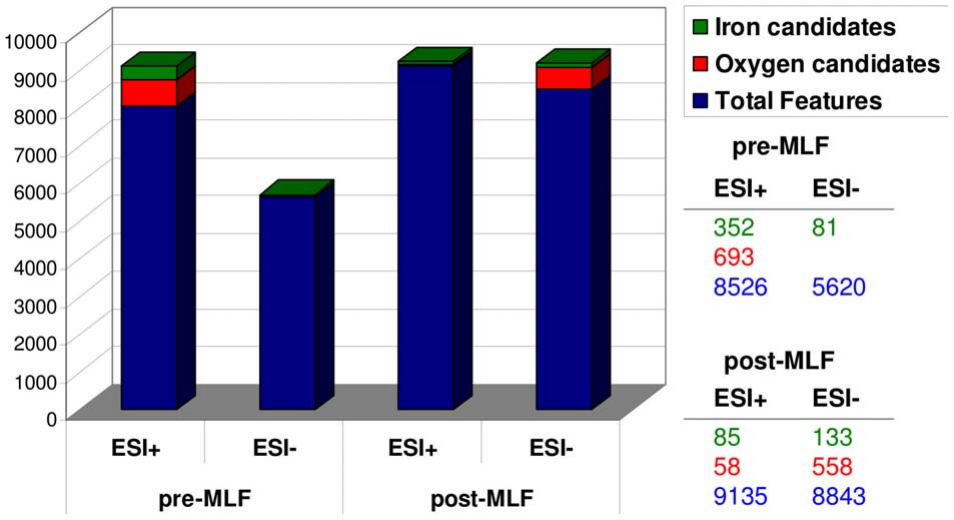
Analysis of UPLC-MS chromatographic data using XCMS, obtained both in ESI positive and ESI negative mode, for the micro-oxygenation experiments before and after malolactic fermentation.

Analysis of UPLC-MS chromatographic data using XCMS revealed 8526 features in ESI positive mode and 5620 in ESI negative mode for the pre-MLF experiments. As regards the post-MLF experiment, the features were 9135 and 8843 for ESI positive and negative modes respectively (Figure 1).

The metabolites formed by bacteria during malolactic fermentation, the metabolites formed by micro-organisms during micro-oxygenation and other metabolites also formed through pure chemical reactions are likely to be the explanation for the higher number of features for the post-MLF experiment. In any case, the numbers of features were comparable to previous wine metabolome studies [19], [25].

### Annotation

**Figure 2:**
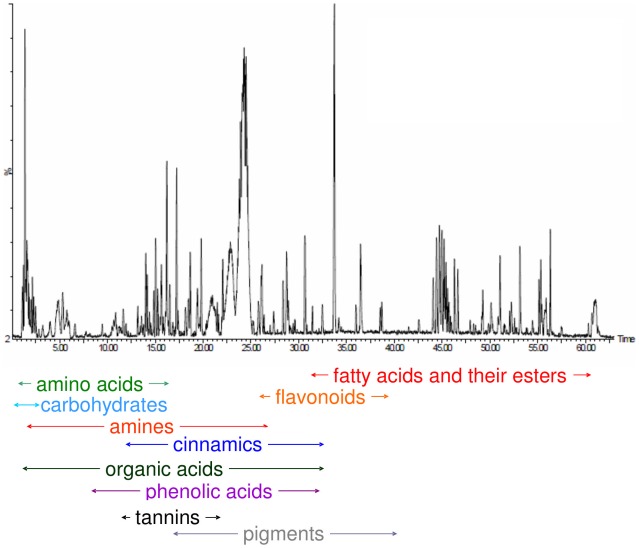
Example of reversed-phase UPLC-ESI-MS chromatogram of a Sangiovese wine. This method can measure in a single run ca. 10 k unique features, associated to both known and unknown wine components, of which over 250 were identified.

Based on the standard data set of our laboratory (chromatographic and spectral libraries of over 400 compounds obtained in the same condition of the experiment) and information from the literature regarding the metabolite profile of wines, it was possible to annotate more than 250 compounds (Table S2). Based on the literature and our group's awareness that wines include a wide range of metabolites with different chemo-physical properties which could be putative biomarkers of the micro-oxygenation procedure, it was decided to run each sample in both positive and negative ESI mode. Over 160 compounds from the ESI positive mode and over 150 from the negative mode were tentatively identified on the basis of both chromatographic and spectral data and of course some of them were identified in both modes. The compounds identified belong to various classes of primary and secondary metabolites, such as pigments, flavonoids, organic acids, phenolics acid, cinnamic derivatives, carbohydrates, amines, amino acids, fatty acids and esters etc. (Figure 2 and Table S2). Mass accuracy for the majority of the annotated compounds was within the widely accepted values for the QTOF MS instruments, thus mass tolerance of 5 ppm (Table S2). In a few cases, when the mass of the compound was too high or its concentration was too small, a larger tolerance was used (10 ppm), by also taking into consideration information from the literature, such as retention time and presence in wines, and at least an extra fragment.

### Untargeted metabolomics

The untargeted LC-MS metabolite fingerprint approach is usually selected because of its sensitivity, resolution and high-throughput capacity, since thousands of compounds can be monitored with a single experiment. To extract useful information from these data, multivariate analysis techniques are used which can handle multiple variables (features) simultaneously and hence also detect important combinations of features. However, the extremely large number of features is a challenge for multivariate data analysis [34]. Further chemometric treatments are therefore frequently employed in LC-MS metabolomics studies to reduce possible drawbacks and to assist with easier identification of biomarkers. An initial application of unsupervised multivariate data analysis to the entire metabolomics data set did not show good group separation. The reason for this may have been that the majority of wine compounds were not affected by our experimental conditions. We therefore first selected the most discriminative features (biomarker candidates) using a supervised SVM classification algorithm.

These selected biomarker candidates were then used to visualise the influence of small oxygen and metal doses variation on wine using unsupervised methods: principal component analysis (PCA) and independent component analysis (ICA).

#### Number of candidate features

Application of the SVM classification algorithm highlighted the candidate features (potential biomarkers) with the largest impact in the different experiments in terms of better group separation in PCA and ICA after the supervised pre-treatment. In the case of metal influence, there were two groups (high and low level); while for oxygen there were four (Table S1).

**Figure 3:**
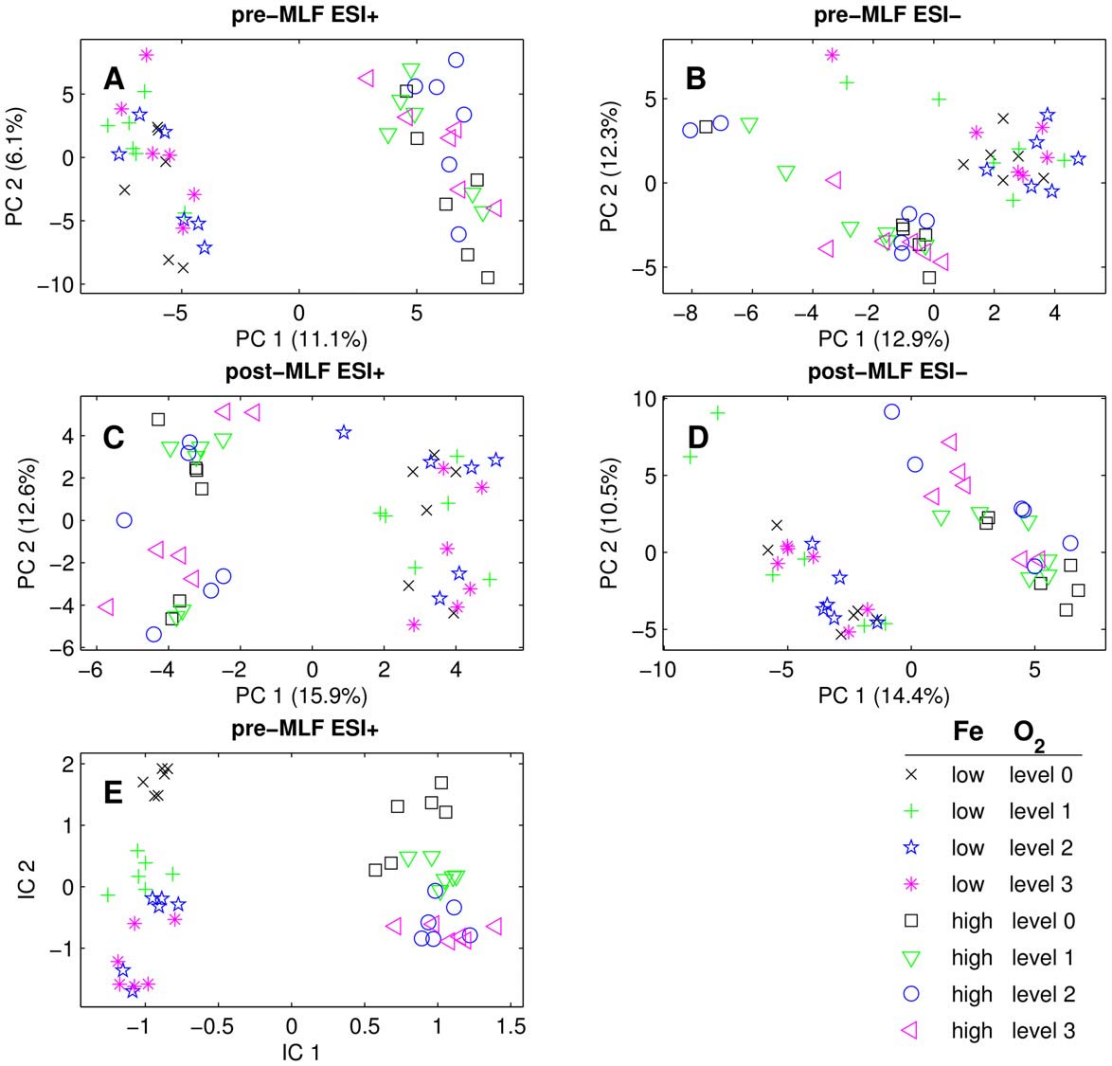
The principal component analysis plots, only applied separately for the candidate biomarkers in each experiment, showed clear group classification of the two metal levels (Panels A–D). In Panel E the candidate biomarkers of both oxygen and metal pre-MLF ESI+ experiments are jointly used to plot an independent component analysis ICA graph. The groups in which no oxygen was added are clearly separated from the others, in the case of both high and low levels of metal.

As shown in Figure 1 in the pre-MLF experiment, 352 features (out of 8526) in the ESI+ analysis and 81 (out of 5620) in the ESI−, showed a significant treatment effect for the two metal levels. The candidate features for the post-MLF experiment were 85 (out of 9135) in ESI+ analysis and 133 (out of 8843) in ESI− (see detailed list with metal level candidate features in Table S3). The PCA plots, only applied separately for the candidate features in each experiment, showed clear group classification of the two metal levels, while this was not possible when all features were included (Figure 3 A–D).

When the influence of the oxygen levels was evaluated, as many as 693 ESI+ features (out of 8526) showed a significant treatment effect in the case of pre-MLF experiments, and the candidates in the post-MLF experiments were 558 ESI- features (out of 8843) and 58 ESI+ features (out of 9135) (Figure 1 and Table S4). The numbers of candidate features extracted from the oxygen experiments were higher, possibly because there were four oxygen levels in each experiment (pre- and post-MLF), double as compared to the metal levels explored. However, the hypothesis that oxygen has a more important effect, affecting the concentration of a greater number of wine metabolites, should not be ruled out.

#### Candidate biomarkers

Annotation of these candidates provided interesting results in two respects.

The candidate biomarkers identified were both compounds known to be influenced by the addition of oxygen and/or metal and also a considerable amount of new information.

#### «Known» candidate biomarkers

In the literature the most studied class of compounds in wine micro-oxygenation are pigments [3], [5]–[10], [14], [35], [36]. Probably the most important and well-known claims in favour of the use of the technique are to enhance red tones and to stabilise the colour in red wine. This is especially important for wines produced from grape varieties containing low amounts and relatively unstable pigments, such as Sangiovese [37].

Sangiovese is nevertheless a grape variety of considerable economic importance globally (area of ∼90 000 ha cultivated), providing some of the most famous wines, such as Brunello di Montalcino and Chianti Classico. Given the pigment's delicacy and reactivity, the level of oxygen introduced could have a positive effect and increase stability or favour reactions, furnishing new and probably more stable forms, or possibly also have a negative effect, though oxidative reactions breaking them down [38].

**Figure 4:**
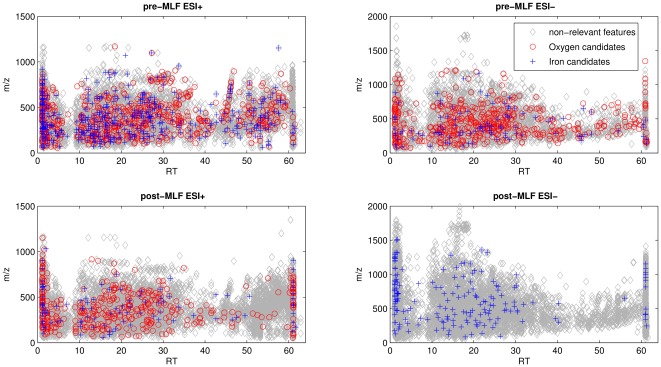
Behaviour of some candidate biomarkers during wine micro-oxygenation performed either before malolactic fermentation (cyanidin 3-glucoside; pyrano-peonidin 3-glucoside, arginine) or after malolactic fermentation (xanthine).

So it was not a big surprise for us to find different pigments among the candidate biomarkers, a result that also indicates the high quality of the experiment. Through this experiment it was possible to reveal the influence of micro-oxygenation not only on the grape anthocyanins (e.g. simple mono-glucosides such as cyanidin 3-glucoside (panel A in Figure 4) and delphinidin 3-glucoside) but also on the pigments formed during wine ageing, such as vitisins (products of the reaction between anthocyanins and acetaldehyde or pyruvic acid e.g. pyrano peonidin 3-glucoside (panel B in Figure 4) and carboxypyrano malvidin 3-glucoside), vinylpheno-pyranoanthocyanins (products of the reaction between anthocyanins and cinnamic acid derivatives, e.g. malvidin 3-glucoside 4-vinylcatechol) and the products of direct or indirect reactions between anthocyanins and flavanols (e.g. peonidin 3-glucoside catechin and malvidin 3-glucoside ethyl-catechin). In total 58 pigments were identified among the candidate biomarkers in the different experiments. More specifically, the number was higher in pre-MLF experiments, in which most of the pigments belonged to the group of flavanol-anthocyanin (direct and ethyl-bridged) derivatives, secondly to the vitisin group and thirdly to the pinotin group. Other studies also carried out on different red wine varieties have demonstrated the greater influence of micro-oxygenation on wine pigments when this is adopted before malolactic fermentation [3], [5], [7], [14], [35]. However, the group of pigments mainly influenced in these studies were vitisins, while there was little information about the reactivity of flavano-anthocyanins derivatives in micro-oxygenation conditions. This difference could be due to the different techniques used in previous experiments (target analyses) as compared to our experiment (untargeted metabolomics), although the most obvious explanation is likely to be the different wine variety used [35], [36]. According to the literature the concentration of these compounds during micro-oxygenation is significantly influenced by the total phenolic content and the pH of the wine [9], [39]. The Sangiovese grape anthocyanin profile is poor in acetylated anthocyanins and is mainly made up of simple monoglucosides. So this characteristic should probably be taken into consideration for the vinification and micro-oxygenation of Sangiovese wines.

Further confirmation that anthocyanins mainly change during pre-MLF conditions could also come from the much smaller number of candidate biomarkers in the ESI+ of post-MLF as compared to pre-MLF experiments (58 as opposed to 693), since red pigments are typically analysed in positive ionization MS conditions, being already positively charged in analytical conditions (Figure 1).

**Figure 5:**
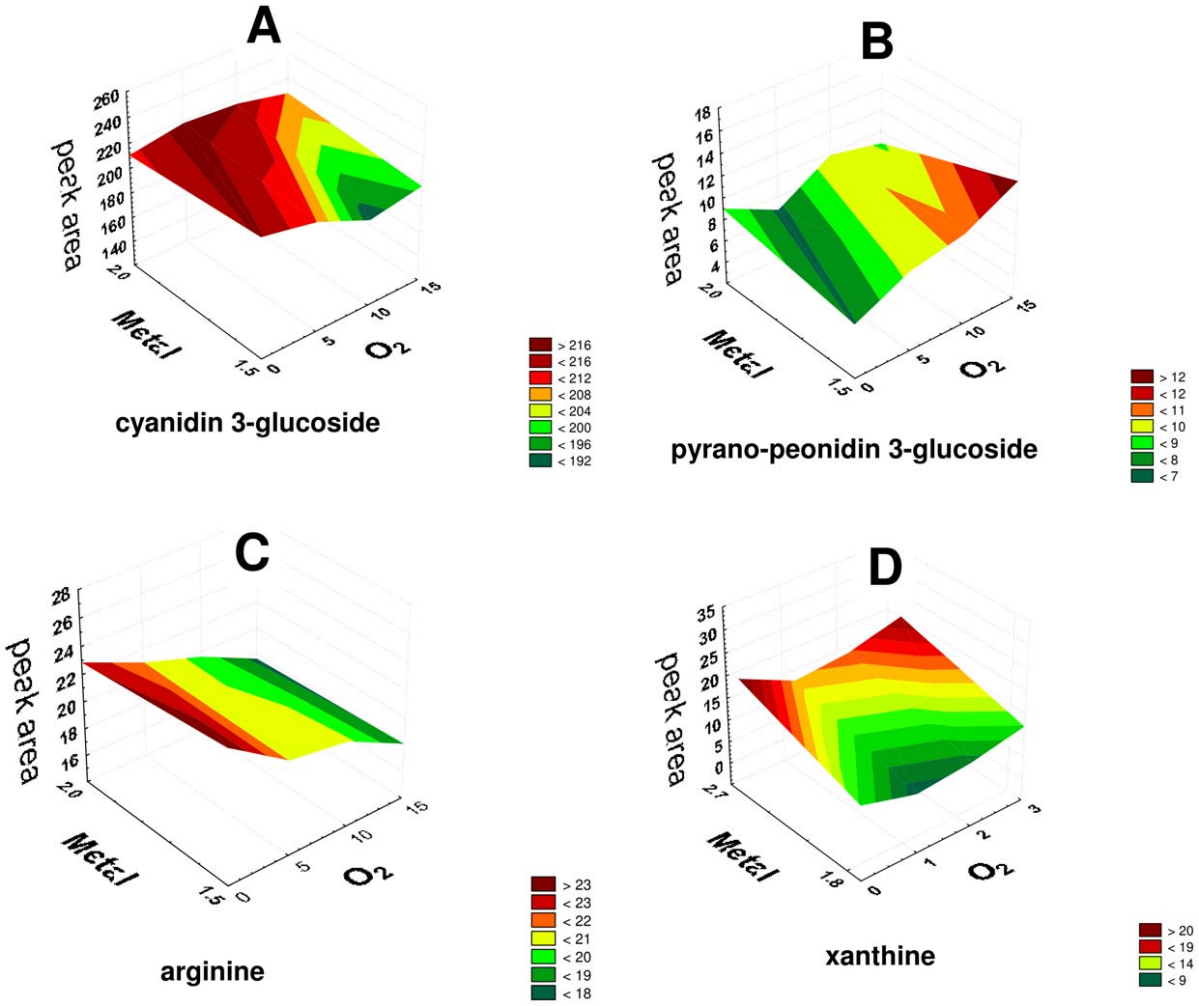
Distribution in the space defined by the *m/z* ratio and chromatographic retention time of the experimental features and candidate biomarkers, obtained both in ESI positive and ESI negative mode, for the micro-oxygenation experiments before and after malolactic fermentation.

Monomeric, dimeric and polymeric flavanols (tannins) are another class of compounds which are considerably influenced by micro-oxygenation, since they not only react with anthocyanins in order to stabilise colour but also react to one another to create new tannins which influence the taste and the structure of the wines [3], [7], [10], [11], [40]. Thus identification of these compounds among candidate biomarkers was expected, both as monomers (*epi*-gallocatechin gallate, *epi*-gallocatechin, *epi*-catechin) and polymers (procyanidins type B and type C). In total 38 tannins from the flavanols class were among the candidate biomarkers in the different experiments. While the number of the pigment candidate biomarkers was greater in the pre-MLF as compared to the post-MLF experiment, for tannins the number of candidate biomarkers was slightly higher in the post-MLF experiment, although the amount of oxygen introduced was much lower. In general the m/z ratio of post-MLF candidate biomarkers was much higher than for pre-MLF (Figure 5). It is known that catechins are polymerized under oxidative conditions in wine and that they build polymers. This polymerization is catalysed by the presence of oxygen in the presence of transition metals and can be blocked by the addition of one anthocyanin molecule to the polymer [3], [8], [9].

So it could be surmised that in our experiment the reactivity of anthocyanins was more clearly expressed during pre-MLF micro-oxygenation, blocking the polymerization of catechins, while during post-MLF micro-oxygenation the number of anthocyanins that could participate in the formation of anthocyanin-flavanol derivatives was limited, so the polymerization of catechins was easier.

#### «Unknown» candidate biomarkers

From the combination of Figures 2 and 5 it is clear that the candidate biomarkers include compounds not only belonging to pigments and tannins. The complete list of candidate biomarkers identified is given in Table S2. The features depending on the level of oxygen introduced and metal levels do not only fall within the zone of pigments and tannins, but are spread throughout the whole chromatographic run and in all m/z values. Hence there are classes of compounds, other than pigments and tannins, that wine science has yet to consider studying in order to evaluate their behaviour in micro-oxygenation conditions.

Among these we identified other classes of phenolic compound, such as caffeic acid, caftaric acid, vanillic acid, gallic acid, quercetin diglucoside, a kaempferol derivative, a taxifolin derivative, miricitrin, ellagic acid and *trans*-piceid. In the light of consolidated knowledge, all *o*-diphenolic compounds may be expected to be sensitive to oxidation and all compounds with *m*-OH groups are nucleophilic and can thus be indirectly involved in oxidative reactions (e.g. add to the intermediates formed by condensation with aldehydes or quinones).

Caffeic acid and its ester caftaric acid are known to be influenced by the presence of iron [12], but in our experiments they were found as candidate biomarkers for oxygen level, both pre-MLF and post-MLF. This finding is probably associated with the participation of these compounds in the formation of vinylphenol-pyranoanthocyanins, which are pigments synthesized from anthocyanins (or other pigments) and cinnamic derivatives. The only identified candidate biomarker from the groups of phenolic and cinnamic acids, found to be significantly influenced by the level of metal was gallic acid. Because of its structural properties (three OH in *ortho* position), gallic acid can react faster with oxygen than other wine polyphenols, and this reactivity is enhanced by the presence of metals such as iron and copper [15]. *trans*-Piceid is a stilbenoid, glucoside or resveratrol, known for its various important biological activities [41]. In our experiment it was a candidate biomarker for iron level. The reactivity of resveratrol and other stilbenoids in wine in micro-oxygenation conditions in the presence of metals has never been studied in detail according to our knowledge. It could differ from that of other polyphenols because of the absence of -OHs in *ortho* position. Since their presence in wines is of considerable importance, because of their putative positive effect on human health, it would be of great interest to study their behaviour during wine micro-oxygenation.

Finally, among the candidate biomarkers there were classes of metabolites never taken into consideration in the bibliography of wine micro-oxygenation. Although wine micro-oxygenation has been extensively studied in the last 20 years, as regards the mechanisms taking place during the application of this technique, there is no information in the literature about its influence on classes of compounds such as primary metabolites (e.g. amino acids and sugars) and also on secondary metabolites such as organic acids, bioamines and fatty acids.

Thus among the candidate biomarkers indentified in this study there were also organic acids, such as succinic acid, lactic acid, malic acid, abscisic acid, indolic acid and gluconic acid; amino acids such as arginine and its metabolite agmantine, proline, histidine, methionine, tryptophan and leucine; fatty acids such as linolenic acid, arachidonic acid, hexenoic acid and oleic acid; oligosaccharides and raffinose; sterols (pomonic acid, pomolic acid, ursolic acid, and stearic acid); and the purine bases xanthine and uridine and some compounds related to their metabolism (uric acid, isoxanthopterin). Moreover, different features were annotated in the post-MLF experiment, indicating fragments of typical wine oligosaccharides [42]–[44].

One of the most interesting results of our study was that among oxygen candidate biomarkers there were also some typical microorganism metabolites. In this experiment, once alcoholic formation ended, the wine no longer offered an anaerobic environment; the micro-organisms stop fermentation and oxidative or respiratory metabolism through the citric acid (TCA) cycle takes place. The addition of a small amount of oxygen should enhance mitochondria TCA metabolism [45]. Products of this metabolism are acetaldehyde and pyruvic acid, which are used in the formation of vitisin and ethyl bridged anthocyanins-catechin derivatives; succinic acid (a candidate biomarker for pre-MLF); and through the *α*–ketoglutarate path, argine and proline (candidate biomarkers for pre-MLF). The amino acid tryptophan and the carbohydrate raffinose (candidate biomarkers for pre-MLF) are typical nutritional factors of this metabolic cycle. In the absence of sugars, yeast can also use the glyoxylate cycle as a respiratory metabolism to synthesize sugars. An important intermediate for this path is succinate, which can also deliver succinic acid (a candidate biomarker for pre-MLF). Arginine (a candidate biomarker for pre-MLF, panel C in Figure 4) is a basic amino acid. In an acidic medium such as wine its guanidinium group has a positive charge which can easily be delocalized, enabling the formation of multiple double bonds and reaction with other active species. Moreover, arginine participates in reactions that generate the biogenic amines responsible for different wine defects. Agmatine is an intermediate for these biosynthetic pathways [46], [47]. Further proof that these amino acids are involved in biosynthetic paths under these conditions comes from the fact that *Saccharomyces* requires oxygen for tryptophan and proline metabolism, while arginine can be metabolised in both aerobic and anaerobic conditions [46], [47].

In the light of these results, new and different hypotheses about the mechanisms occurring during wine micro-oxygenation could be proposed. Acetaldehyde and pyruvic acid, products of micro-organisms respiratory metabolism, react with anthocyanins to form vitisins [3]. Of course acetaldehyde and pyruvic acid could also be produced in wines by Fenton oxidation respectively of ethanol or malic and lactic acids [13], but still there are not conclusive evidences if the ones participating to the formation of vitisins are products of chemical reactions or/and of wine microbiota metabolism. This issue could be an interesting subject of further investigation.

In this experiment we noticed that acetaldehyde and vitisins increased in pre-MLF conditions, while the simple monoglucosides of anthocyanins decreased, when increasing the level of the oxygen in the wine (Table S1 and Figure S3). In the micro-oxygenation treatments carried out after malolactic fermentation, grape anthocyanins continued to decrease, probably because of their low stability in post-MLF conditions, while vitisins decreased, maybe also because Lactic bacteria consume acetaldehyde, while the polymerization of tannins was more clearly expressed as compared to pre-MLF experiments. Tannin polymerization is enhanced by the presence of oxygen and can be blocked by the addition of an anthocyanin [3]. Tannins are generally regarded as inhibiting the growth of microorganisms. The monomers with intracellular mechanisms, the polymers by extracellular binding with proteins or polysaccharides [48]. Some bacteria tolerate polymeric tannins using the mechanism of extracellular secretion of oligosaccharide, which could explain why both oligosaccharides and tannins of higher m/z as compared to the pre-MLF experiment were found among the candidate biomarkers in the post-MLF experiment. It is possible to speculate that in wine the reaction of grape anthocyanins with acetaldehyde could be also reversed, since acetaldehyde could also be very easily cleaved from the vitisin structure, providing an anthocyanin with a C4 position of much higher reactivity as compared to the flavylium cation form. However, further investigation is needed in order to verify the reversibility of this reaction.

**Figure 6:**
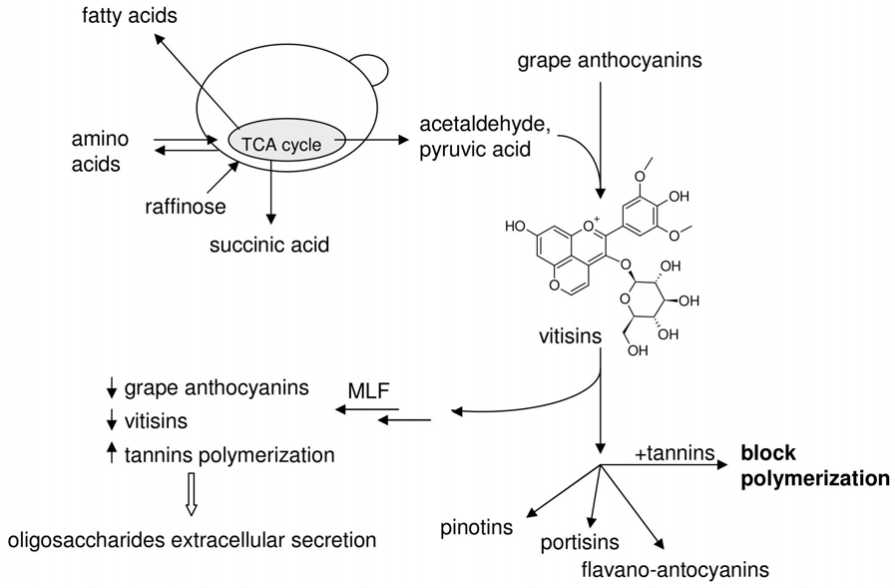
General scheme suggesting a link between the biomarkers of wine micro-oxygenation, involving both a change in concentration of TCA cycle metabolites and the formation and interconversion of wine pigments during wine micro-oxygenation.

Therefore, it could be surmised that microorganisms try to regulate the polymerization of tannins through the respiratory metabolism and that vitisins are intermediates in the path of more stable pigments (Figure 6).

The formation of vitisins could play the dual role of enhancing colour stability, since vitisins are more stable than grape anthocyanins, while on the other hand increasing anthocyanin reactivity towards the reaction delivering new pigments. This observation is in total agreement with a recent work where vitisins are considered intermediate for the formation of vinylpyranoanthocyanin-flavanols in Port red wine [49]. It is worth recalling that a metabolomic study of the fate of wine and grape polyphenols in humans also showed that a grape-red wine mix extract (but not the pure grape extract) had an impact on mitochondrial TCA cycle turnover [28]. Even if this observation was obviously not reflected in wine ageing conditions, the influence of iron in the TCA cycle induced by wine micro-oxygenation practices should not be overlooked. After many years of research aimed at establishing suitable analytical tools for the control of micro-oxygenation, nowadays organoleptic testing of wine is still considered the only reliable method. Until now, the results of most studies were unsuitable for application to industrial practice, relying on sophisticated and expensive analytical methods to measure tannins and pigments. So an alternative perspective to approach this unsolved problem could possibly be to focus on the analysis and measurement of TCA cycle metabolites, such as arginine, proline or succinic acid. What is more, amino acids and succinic acid are precursors of multiple metabolites with both a positive and a negative influence on the organoleptic character of wines. Furthermore, metabolites in this group have been reported to have strong correlation with oxygen doses in different wine micro-oxygenation studies [3], [7], [8]. Since these studies were based on different wine varieties, it could be suggested that these changes are of general value, as they are not variety-dependent, thus supporting the TCA hypothesis.

Iron levels also provided some interesting new results as far as unknown candidate biomarkers are concerned. Xanthine, an alkaloid and purine base, was one compound that had the clearest and strongest correlation with the level of metal in the pre-MLF experiment (panel D in Figure 4). The fact that xanthine oxydase, an enzyme that converts hypoxanthine to uric acid via xanthine, has an iron/sulphur redox centre, could explain this phenomenon [50]. One of the microorganism's defence strategies under oxidative stress is the activation of xanthine oxidase to produce uric acid, which has powerful antioxidant properties [51]. The fact that uric acid was also a candidate biomarker for the same data set strengthens the possibility that this reaction took place during our experiment.

From the literature, iron is known to play a catalytic role in the oxidation pathways of polyphenolic compounds. In Figure 3E the candidate biomarkers of both oxygen and metal pre-MLF ESI+ experiments are jointly used to plot an ICA graph. The groups in which no oxygen was added are clearly separated from the others, in the case of both high and low levels of metal. On the other hand, the groups with the addition of oxygen are more clearly separated in the experiments with the lowest level of metal. Since metal acts as a catalyst, when used at the higher level in our experiment, it had a greater influence than oxygen and forced the reaction within a specific pathway. When the level of iron was lower, oxygen had a clearer effect.

### Concluding remarks

Over the years, various research projects have tried to understand and monitor the mechanism occurring when wine micro-oxygenation is applied, through target analysis. In this study, untargeted UPLC-QTOF MS analysis coupled with multivariate statistics was used to study this globally applied practice. The results of our study made it possible to confirm most of the known wine micro-oxygenation biomarkers reported in the literature, within a simple set of experiments, these mostly being red pigments and tannins. Moreover, it revealed new chemical changes in the Sangiovese wine profile, when subjected to various oxygen levels in the presence of two different levels of iron. A number of additional primary and secondary metabolites, not considered in previous studies, appear to be candidate biomarkers. These new candidate biomarkers, and especially those showing a biphasic effect in presence of variable levels of oxygen, should be further studied and validated for a comprehensive understanding of the changes occurring in the wine metabolite fingerprint during the micro-oxygenation process. This information is much needed, since it could be useful for developing analytical tools able to assist winemakers with more appropriate control of the micro-oxygenation process.

## Materials and Methods

### Micro-oxygenation

Red wine from the *Vitis vinifera* L. cv Sangiovese grape variety (harvested at optimal maturity and in good conditions in 2009) was produced at the Consorzio Tuscania (Tuscany – Italy) experimental winery, following widespread winemaking methodology. Following alcoholic fermentation, 240 hl of wine were homogeneously distributed in 24 stainless steel 10 hl tanks, in order to run 8 triplicate 7 week experiments.

Tanks were kept full and saturated with the inert gas Argon. SAEN 5000 (Parsec s.r.l., Florence, Italy) micro-oxygenation equipment was used for oxygen dosage, using ceramic plate diffusers suitable for small tanks. Temperature was controlled and kept at 18°C throughout the period of the experiment.

The micro-oxygenation experiment before malolactic fermentation (pre-FLM) involved two levels of iron (∼1.3 and 1.7 mg/L), with four different levels of oxygen (0, 5, 10 and 15 mg/L/month) being introduced during seven weeks. Detailed experimental data are given in Table S1.

Malolactic fermentation took place following this first micro-oxygenation treatment and the wine was then pooled, in order to provide a homogenous mass, and once again redistributed in the 24 tanks for a second micro-oxygenation treatment. The post malolactic fermentation experiment (post-MLF) involved two levels of iron (1.7 and ∼2.7 mg/L Table S1), with four different levels of oxygen (0, 1, 2 and 3 mg/L/month) introduced during three months.

At the end of both experiments (pre-MLF and post-MLF) a homogeneous sample of each wine obtained was bottled in 375 mL dark glass wine bottles and kept at 4°C, until analysis. All the samples were analysed together, less than 3 weeks after the end of each experiment (pre-MLF and post-MLF). All the biological replicates were analysed twice for each ESI-MS condition. All micro-oxygenation and metal levels were within typical vinification levels, without any extremes. The levels of dissolved oxygen were measured daily using oxo-luminescence with PreSens (Nomacorc) in all wines and were always found to fall within the concentrations typical for microoxygenation treatments (Figures S1 and S2). The amount of acetaldehyde was measured regularly using GC in all wines and was found to increase according to the level of oxygen applied and the duration of the treatments (Figures S3 and S4). Free and total sulphur dioxide were regularly measured by iodometric titration and results are summarised in Table S1.

### Chemicals

The water used was purified in Milli-Q. Iron(II) sulphate hydrate and all other chemicals used in this study were of the highest purity grade available and purchased from Sigma-Aldrich (St. Louis, MO), unless otherwise stated. Pure, HPLC grade (+)-catechins, (−)-epicatechin, procyanidin B1, procyanidin B2, procyanidin B4, procyanidin A2, quercetin 3-glucuronide, D-talose, D-arabitol, daidzein, syringetin 3-glucoside, syringetin 3-galactoside, syringetin, quercetin, quercetin rutinoside, quercetin arabinoside, myricetin, quercetin 3,4-diglucoside, laricitrin, kaempferol, keampferol 3-glucoside, kaempferol 3-glucuronide, isorhamnetin 3-rutinoside, isorhamnetin and isorhamnetin 3-glucoside were obtained from Extrasynthese (Genay, France). Delphinidin 3-glucoside, cyanidin 3-glucoside, petunidin 3-glucoside, peonidin 3-glucoside, and malvidin 3-glucoside were from Polyphenols Laboratories AS (Sandes, Norway).

### UPLC-QTOF MS

A Waters Acquity UPLC coupled via an electrospray ionization (ESI) interface to a Synapt HDMS QTOF MS (Waters, Manchester, UK) operating in W-mode and controlled by MassLynx 4.1 was used. All samples were analysed on a reversed phase (RP) ACQUITY UPLC 1.8 µm 2.1×150 mm HSS T3 column (Waters) at 30°C with gradient elution starting isocratic from 100% A (water, 0.1% formic acid) from 0 till 6 min and then increasing linearly over 56 min to 100% B (methanol, 0.1% formic acid) where it was held isocratic until 60 min, with 0.3 ml/min flow rate. Injection volume was 5 µL and the samples were kept at 4°C throughout the analysis [52]. The in-batch order of all samples analysed in this study was random.

Mass spectrometric data were collected by different runs in positive and negative ESI mode over a mass range of 50 to 3000 amu with scan duration of 0.3 s in centroid mode and in low and high energy. The transfer collision energy and trap collision energy were set at 6 V and 4 V for the low and 30 V and 6 V for high energy acquisition respectively. The source parameters were set as follows: capillary 3 kV for positive scan and 2.5 kV for negative scan, sampling cone 25 V, extraction cone 3 V, source temperature 150°C, desolvation temperature 500°C, desolvation gas flow 1000 L/h and nebulizer gas 50 L/h. External calibration of the instrument was performed at the beginning of each batch of analysis by direct infusion of a sodium formate solution (10% formic acid/0.1 M NaOH/Acetonitrile at a ratio of 1/1/8) by controlling the mass accuracy from 40 to 2000 m/z (less than 5 ppm) and mass resolution (over 14000 FWHM). LockMass calibration was applied using a solution of leucine enkephaline (0.5 mg/L, m/z 556.2771 for positive and 554.2620 for negative ion mode) at 0.1 mL/min.

Wines were uncorked under nitrogen atmosphere and an aliquot was transferred after filtration with 0.2 µm PTFE filters into a 2 mL autosampler amber vial (filled to its capacity) and injected without any further pre-treatment.

A mix of eleven compounds comprising *trans*-resveratrol, caffeic acid, ellagic acid, catechin, quercetin, quercetin-3-glucoside, cyanidin-3-galactoside, methyl stearate, 4-aminobutyric acid, glutathione and ursolic acid in methanol/water (70/30) was used as quality control standard sample (STDMIX). STDMIX samples were injected after every seven wine samples during analysis.

The complete data sets of features identified by the XCMS software are available as text files for download in our site (http://cri.fmach.eu/QA/Wine-Micro-oxygenation). Also the CDF files (∼10 G) are available after request due to the large amount of raw data.

### Data analysis

Raw data was converted to CDF (Waters Databridge) format and then processed using XCMS [53], [54] for feature extraction, grouping and alignment. For peak picking, the matched Filter algorithm was used with a peak width estimate of 20 s. The signal to noise parameter was set to S/N = 6. Data extraction parameters were selected on the basis of the characteristics of chromatographic and mass spectrometric performance [52]. To characterise potential groups of mass/retention features (fragments, isotopes and adducts), we applied the R package CAMERA [54], using the default settings, including a mass tolerance of 10 ppm; window width (perfwhm) 0.7; and peak correlation threshold (cor_eic_th) 0.75.

Peak annotation was performed manually by comparing retention times and mass spectra (mass difference less than 10 ppm of experimental value) to those of the standard, when available. Tentative identification of the chromatographic peaks, without a standard, was made using the spectral features (mass difference less than 10 ppm of theoretical value and at least one indicative fragment) and using the data in the literature. Integration of all annotated compounds was performed using the TargetLynx utilities of MassLynx Waters Software. Candidate biomarkers for separation of the various levels of metal and oxygen were identified by using a Support Vector Machine (SVM) algorithm and visualised subsequently using principal component analysis (PCA) and independent component analysis (ICA). Data analysis was performed using R and Matlab.

The support vector machine (SVM) algorithm [55], [56] is a modern classification algorithm, extensively applied within the field of molecular biology, including the field of metabolomics [57]–[59]. The high classification performance of a SVM is achieved by identifying a discriminative hyperplane of largest distance to the sample group thereby performing a low generalisation error. Standard cross-validation is used to optimise the SVM settings. In contrast to other discriminant analysis techniques, a SVM does not require normally distributed data, and hence is very suitable for most experimental data. To identify the features with the highest impact on the separation of metal and oxygen levels, we used linear SVM applied to variance normalised data.

Similar to loadings in unsupervised PCA, linear SVM provides a weight for each feature according to its importance in the classification. The features with the largest impact provided us with a list of biomarker candidates which were subsequently used in PCA and ICA visualisations.

Classical PCA is a standard technique for visualising and interpreting metabolite data. However, in order to extract better components, we also applied a modern alternative: independent component analysis (ICA). Due to its independence, ICA can provide more meaningful components than PCA. The concept of ICA was first proposed by Comon [60], with subsequent developments by Bell and Sejnowski [61].

ICA has become important for biomedical applications, including gene expression [62], [63] and metabolite profile analysis [64].

## Supporting Information

Figure S1 **Average levels of dissolved oxygen (µg/L) measured at six time points using oxo-luminescence with PreSens (Nomacorc) in all wines during the experiment before malolactic fermentation.** Concentration values were always found to fall within the concentrations typical for microoxygenation. The vertical bar shows the standard deviations.
(TIF)

Figure S2 **Average levels of dissolved oxygen (µg/L) measured at four time points using oxo-luminescence with PreSens (Nomacorc) in all wines during the experiment after malolactic fermentation.** Also in this case the concentration values never exceeded the concentrations typical for microoxygenation. The vertical bar shows the standard deviations.
(TIF)

Figure S3 **Average amount of acetaldehyde (mg/L), measured at five time points using GC in all wines during the experiment before malolactic fermentation.** The concentration of acetaldehyde was found to increase according to the level of oxygen applied and the duration of the treatments. The vertical bar shows the standard deviations.
(TIF)

Figure S4 **Average amount of acetaldehyde (mg/L), measured at four time points, using GC, in all wines during the experiment after malolactic fermentation.** Also in this experiment the concentration of acetaldehyde was found to increase according to the level of oxygen applied and the duration of the treatments. The vertical bar shows the standard deviations.
(TIF)

Table S1 **Experimental design of the two micro-oxygenation experiments, performed respectively before (pre-MLF) and after malolactic fermentation (post-MLF).**
(XLS)

Table S2 **Annotated Metabolites Data.**
(XLS)

Table S3 **Iron level candidate biomarkers.** In yellow are marked diagnostic ions of the identified candidates. In red are marked diagnostic fragments of oligosaccharides.
(XLS)

Table S4 **Oxygen level candidate biomarkers.** In yellow are marked diagnostic ions of the identified candidates.
(XLS)
