# Supplementary figures and images for: A Metabolomic Approach to the Study of Wine Micro-Oxygenation
Source: PLoS One. 2012 May 25;7(5):e37783. doi: 10.1371/journal.pone.0037783 (PMC3360592; doi:10.1371/journal.pone.0037783)

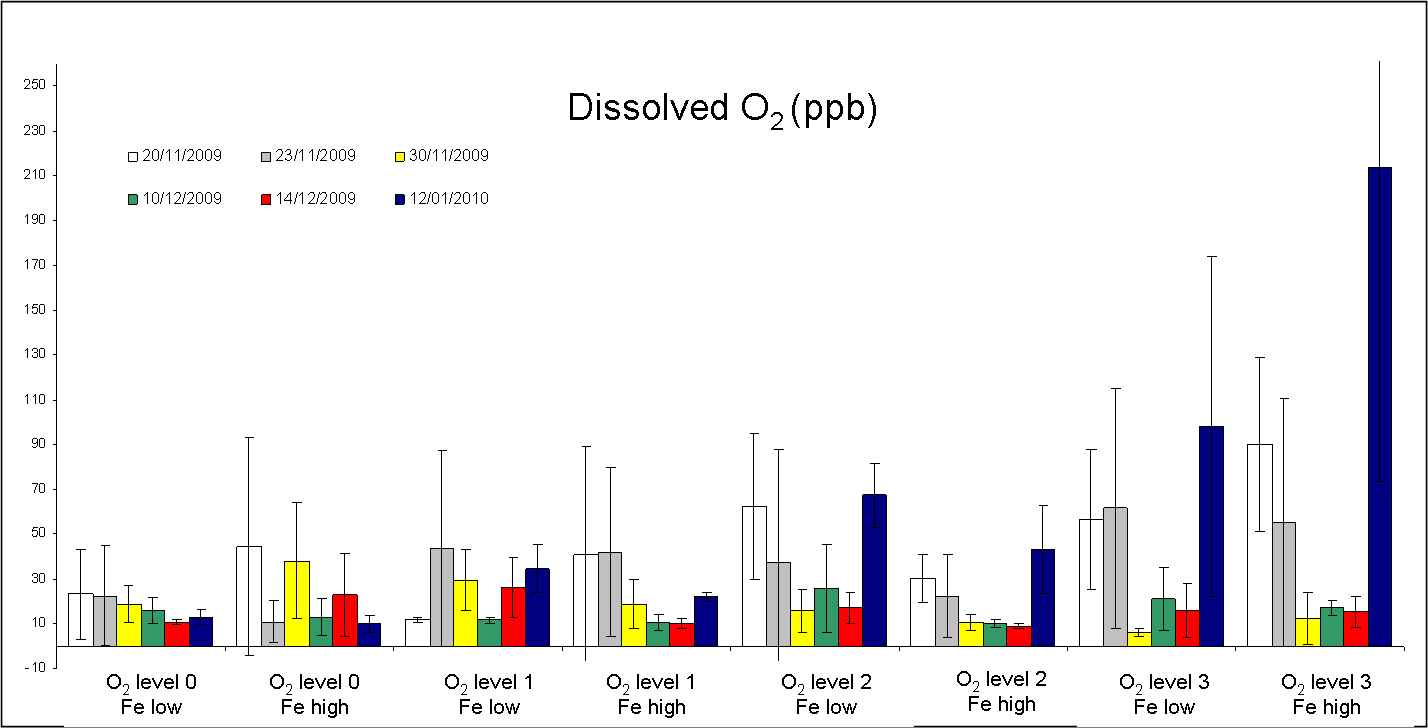

Supplement: Figure S1 — Average levels of dissolved oxygen (µg/L) measured at six time points using oxo-luminescence with PreSens (Nomacorc) in all wines during the experiment before malolactic fermentation. Concentration values were always found to fall within the concentrations typical for microoxygenation. The vertical bar shows the standard deviations. (TIF) [file pone.0037783.s001.tif]

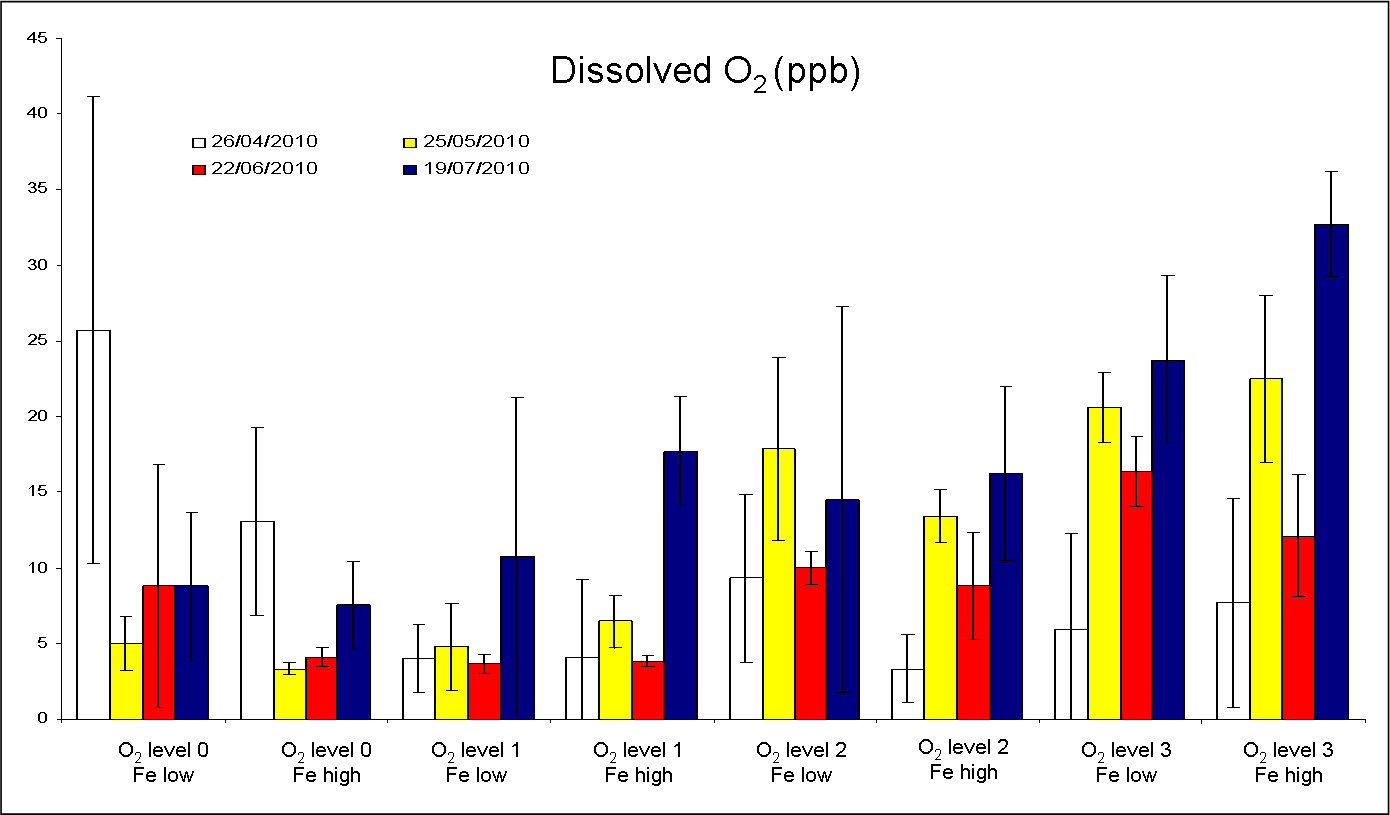

Supplement: Figure S2 — Average levels of dissolved oxygen (µg/L) measured at four time points using oxo-luminescence with PreSens (Nomacorc) in all wines during the experiment after malolactic fermentation. Also in this case the concentration values never exceeded the concentrations typical for microoxygenation. The vertical bar shows the standard deviations. (TIF) [file pone.0037783.s002.tif]

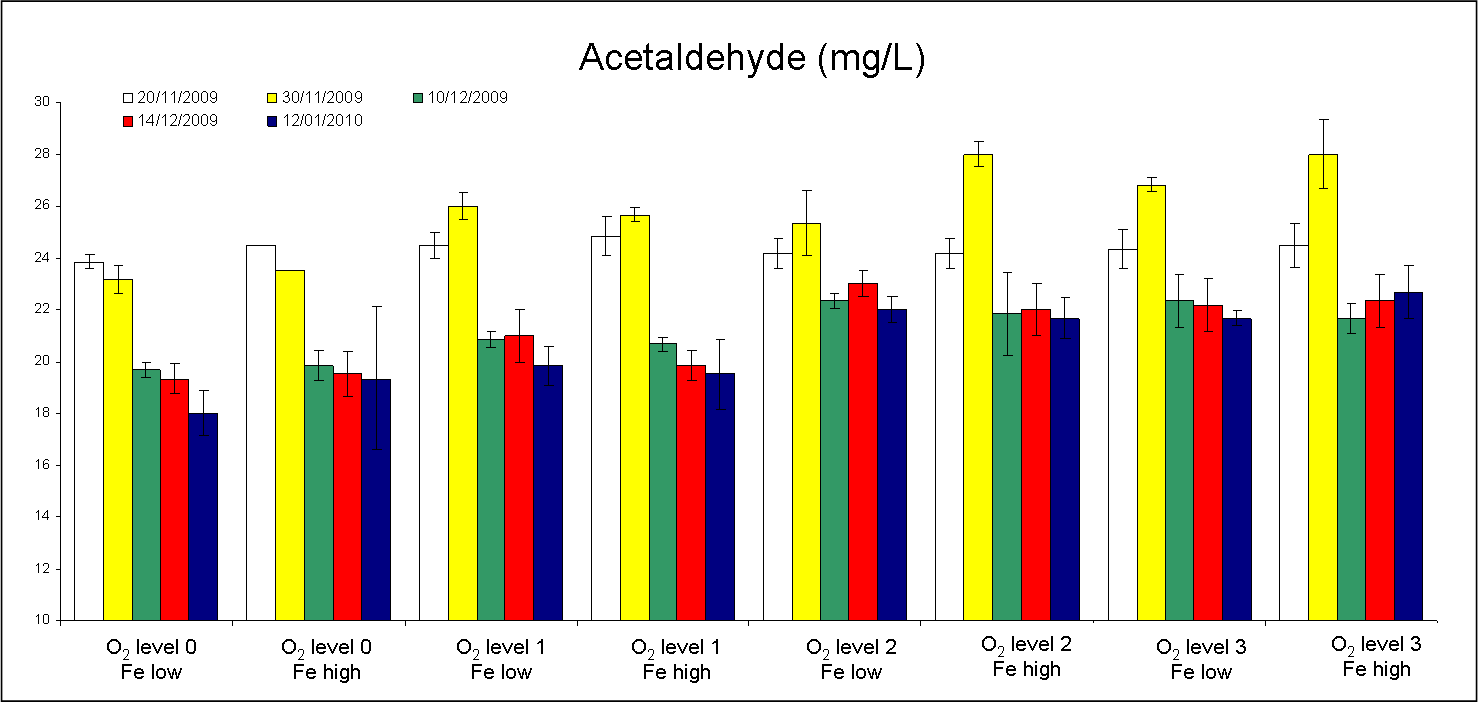

Supplement: Figure S3 — Average amount of acetaldehyde (mg/L), measured at five time points using GC in all wines during the experiment before malolactic fermentation. The concentration of acetaldehyde was found to increase according to the level of oxygen applied and the duration of the treatments. The vertical bar shows the standard deviations. (TIF) [file pone.0037783.s003.tif]

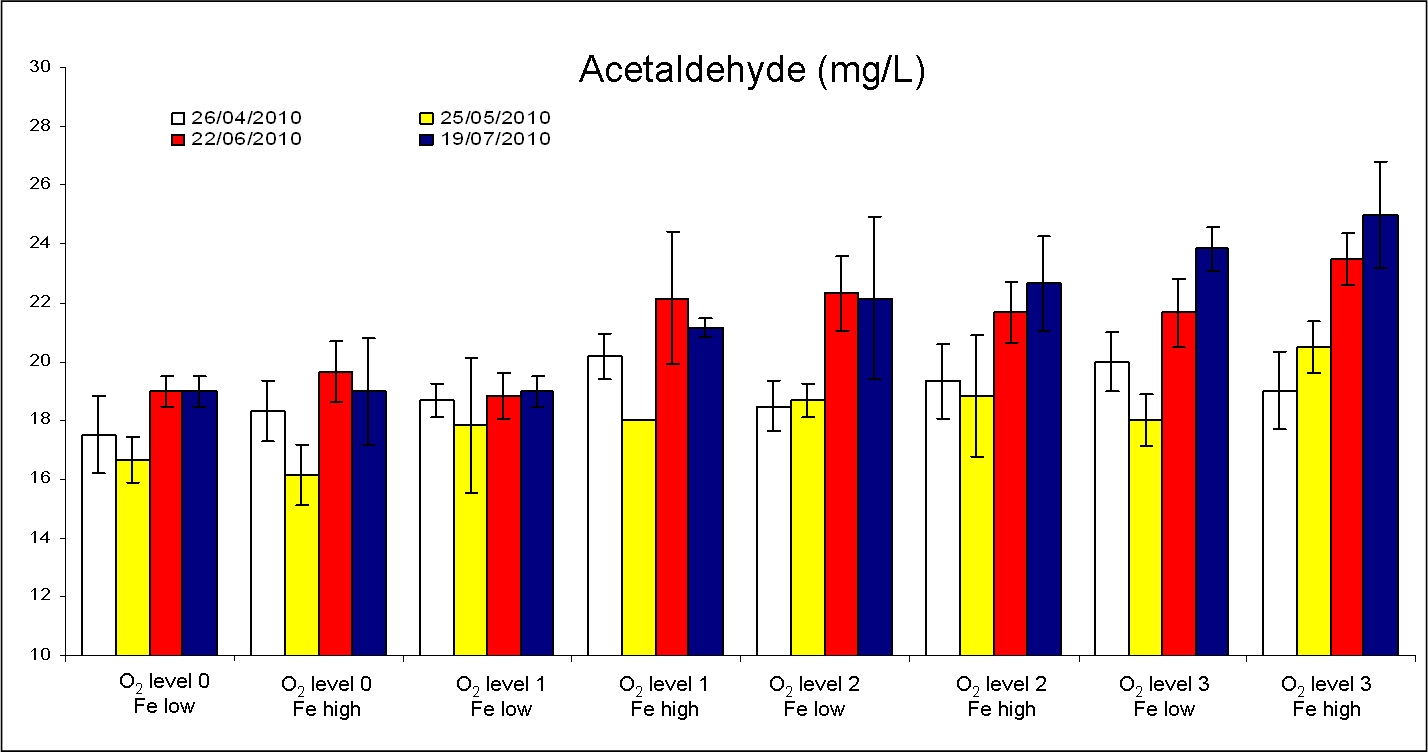

Supplement: Figure S4 — Average amount of acetaldehyde (mg/L), measured at four time points, using GC, in all wines during the experiment after malolactic fermentation. Also in this experiment the concentration of acetaldehyde was found to increase according to the level of oxygen applied and the duration of the treatments. The vertical bar shows the standard deviations. (TIF) [file pone.0037783.s004.tif]
